# Dental Superheroine: A Culturally Adapted Storytelling Strategy for Oral Health Education in Rural Peru

**DOI:** 10.1016/j.identj.2025.103867

**Published:** 2025-08-29

**Authors:** Yuly Ruby Arce-Alva, Heber Isac Arbildo-Vega, Rubén Arturo Aguirre-Ipenza, Oscar Pizarro-Salazar, Franz Tito Coronel-Zubiate, Lenin Edwads Velez-Rodriguez, Carlos Alberto Farje-Gallardo

**Affiliations:** a Escuela Profesional de Psicología, Universidad Nacional Toribio Rodríguez de Mendoza de Amazonas, Chachapoyas, 01001 Perú; b Escuela de Estomatología, Universidad San Martín de Porres, Chiclayo, 14012, Peru; c Facultad de ciencias de la salud, Universidad Continental, Lima, 15046, Peru; d Escuela de Estomatología, Universidad Nacional Toribio Rodríguez de Mendoza de Amazonas, Chachapoyas, 01001, Chachapoyas, Perú

**Keywords:** Dental superheroine, Oral health, Cultural adaptation, Health promotion, Fictional characteristics, Community participation

## Abstract

**Introduction and aims:**

Oral health problems remain a serious concern in rural areas of underdeveloped countries, where high rates of dental caries and periodontal disease are worsened by limited access to health education. This study evaluated the effectiveness of a culturally adapted dental superheroine to promote oral hygiene practices by encouraging oral health education.

**Methods:**

This study was conducted in two phases. The first phase involved designing the character and adapting it culturally through stakeholder interviews and qualitative analysis. In the second phase, the superheroine was publicly introduced during a community event, followed by a structured survey of 300 participants to assess public perception and cultural identification.

**Results:**

Overall, 82% of the respondents had a very positive perception of the superheroine, and 89.67% of the participants rated the initiative as positive. A significant correlation was found between cultural engagement and positive perception (r = 0.749, *P* < .05), whereas a regression analysis indicated that 56.1% of the variance in positive perception could be attributed to the level of cultural engagement (R^2^ = 0.561).

**Conclusions:**

The dental superheroine model represents a constructivist approach that integrates education with local culture. Its scalability and adaptability make it a promising strategy for implementation in similar contexts.

**Clinical relevance:**

This study highlights the importance of adapting public health strategies to local traditions and customs, which could improve the effectiveness of interventions in rural settings where traditional strategies may not be as effective.

## Introduction

Oral health is a fundamental component of overall well-being, directly influencing quality of life and social interactions.^1^ Despite its importance, the city of Chachapoyas, like several other regions, faces persistent challenges in promoting effective oral hygiene practices. Although public and private health entities have made efforts to improve oral hygiene, significant gaps remain in access to both resources and information.^2^ Limited dental care infrastructure and scarce resources further exacerbate these challenges, contributing to the continued prevalence of oral health problems within the community.^3^^,^^4^

Located in the Andean region of Peru, Chachapoyas experiences a high burden of oral health problems. Recent data indicate that 90.4% of the population suffers from dental caries, 85% from periodontal disease, and approximately 70% from malocclusion.^5^ A shortage of oral health professionals and limited access to preventive care have led to a high incidence of untreated conditions, which negatively impact individual productivity and social well-being. Ramos-Gomez et al.^6^ reported that untreated oral diseases can significantly impair physical health and quality of life, particularly in underserved communities. Similarly, Natassa et al.^7^ argued that addressing these challenges requires culturally tailored approaches that extend beyond traditional health interventions.

In response to such challenges, this study explores the introduction of a fictional dental superheroine as an innovative strategy to engage the community in oral health promotion. Research suggests that fictional characters can be effective tools in health education, especially in rural and marginalised communities where conventional health campaigns may lack cultural resonance.^8^^,^^9^ When designed to reflect local values and identities, these characters can serve as relatable role models, capturing attention and effectively conveying health messages.^10^^,^^11^ This study aims to harness that potential by embedding oral health messages into the cultural fabric of Chachapoyas, thereby fostering a collective commitment to improved oral hygiene.

Numerous studies have emphasised the positive influence of fictional characters on public health attitudes and behaviours. Superhero figures, for instance, have been shown to promote healthy eating and hygiene habits in children, demonstrating their potential as tools for behavioural intervention.^12^^,^^13^ Moreover, culturally tailored educational strategies have been shown to enhance message retention and support long-term behaviour change.^14^ These findings underscore the importance of aligning health education initiatives with the cultural values of target populations.

The educational approach underpinning this study aligns with constructivist principles, emphasising the active participation of individuals and communities in the learning process. By developing a culturally resonant character, the intervention seeks to create meaningful connections between oral health messages and the cultural context of Chachapoyas. Constructivist theory posits that knowledge is built through interaction with one’s environment, and this study leverages local cultural identity to enhance the relevance and impact of its messaging.

Moreover, the incorporation of interactive elements, such as 3-dimensional (3D) prototypes and participatory educational activities, promotes experiential learning, a core tenet of constructivist pedagogy. These components encourage active engagement, particularly among younger audiences, fostering deeper understanding and ownership of oral health practices. This participatory approach not only strengthens knowledge retention but also nurtures a community-wide commitment to sustainable, transformative health behaviours. The objective of this study is to design and implement a culturally resonant dental superheroine as a constructivist tool to promote oral health in Chachapoyas. This includes the identification of essential elements that enhance cultural engagement and foster active participation in adopting positive oral health practices. To achieve this objective, the study was carried out in two main phases: the first phase focused on characterising the dental superheroine through cultural adaptation and stakeholder input, and the second phase involved the development of the superheroine concept and its dissemination within the community.

## Methods

### Study design

The study was structured in two main phases: the characterisation of the dental superheroine and the development of the final implementation strategy. Each phase had specific objectives and employed customised methods and materials to ensure the validity and applicability of the results.

***Phase 1: Characterisation of the dental superheroine***

An in-depth study was conducted to analyse the cultural and social factors shaping the perceptions of fictional characters, assessing their potential as educational tools. In this phase, key characteristics were identified for the dental superheroine that would foster cultural acceptance and resonate with the community, ensuring the effectiveness of her message.^15^ First, a comprehensive literature review was conducted to examine the previous research on the impact of fictional characters on health education^16^ and awareness campaigns in a similar context.^17^ Second, interviews were conducted with local leaders in oral health, including the Dean of the College of Dentists of Peru—Chachapoyas Chapter, the director of the Professional School of Stomatology of the National University Toribio Rodriguez de Mendoza of Amazonas (EPEST-UNTRM), educators and community members. These professionals, all based in Chachapoyas, evaluated the feasibility of the proposal and helped to define the key attributes of the character.

Based on these findings, the team decided that the main character should be female, a choice that aligns with prominent local figures, such as Matiaza Rimachi, the heroine of the war of independence against the Spanish,^18^ and Virgen Asunta, the local patron saint of the Catholic Church.^19^ The character was designed with youthful and dynamic features that embody traditional Chachapoyan culture, including fair skin, brown hair and eyes and an athletic build.^20^ The character is equipped with a shield symbolising protection, which is also incorporated into the emblem of the Professional School of Stomatology. The shape of the shield was selected to represent part of the oral opening scheme, and the character wore a mask to maintain anonymity. The choice of these attributes was made deliberately to appeal to a younger audience and to encourage positive identification with the character.

***Phase 2: Proposal development***

Guided by a qualitative analysis, the dental superheroine was developed and introduced through targeted dissemination strategies, enabling an assessment of her impact on community awareness and behaviour. The character was designed, implemented and evaluated as part of a broader initiative to promote oral health in Chachapoyas, following the guidelines established during the characterisation phase.^21^ In addition, a range of educational materials was created to accompany the superheroine during dental health campaigns and institutional parades. These materials included toothbrushes, tooth models, brochures and posters. The official presentation of the dental superheroine took place on 16 September 2024, during the institutional anniversary parade of the Universidad Toribio Rodríguez de Mendoza de Amazonas (UNTRM), providing an opportunity for initial impact evaluation through surveys of the attendees.

**Fig. 1:**
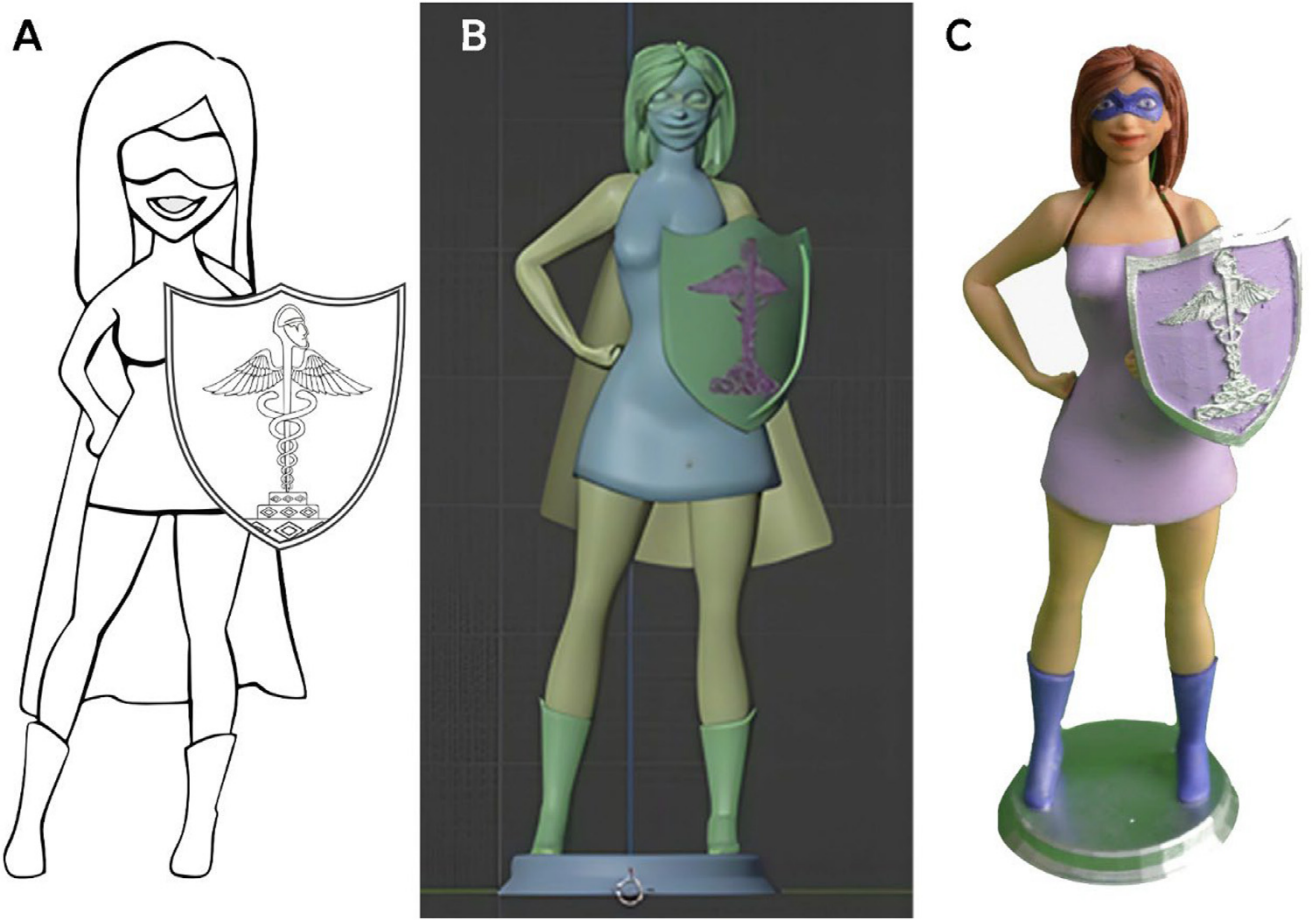
Development of the dental superheroine.

The character development process began with a hand-drawn pencil sketch as the initial concept (Figure 1A). Following team feedback, the design was digitally rendered by the graphic designer using Blender 3D software, resulting in the first digital version of the character.^22^ This digital model was subsequently reviewed, and after the final adjustments, the design was approved by a consensus among all the team members (Figure 1B).

The next step consisted of the fabrication of the 3D prototypes, which were materialised via a CR-10S 3D printer with polylactic acid (PLA) filaments. After the prints were obtained, the team chose the final prototype, which was then coloured by a painting professor from the Escuela Superior de Formación Artística Pública. This artist, operating in accordance with the constructivist principles that advocate for the utilisation of tangible and sensory resources to enhance learning outcomes, incorporated culturally resonant symbols into the design of the superheroine. This incorporation served to deepen the emotional and cognitive engagement of the participants, and thus render the health messages in a more memorable and impactful manner. The team unanimously approved the final prototype (Figure 1C).

#### *Evaluation of qualitative insights*

The qualitative evaluation of the project was integral to understanding the cultural and pedagogical resonance of the dental superheroine initiative. Semi-structured interviews and focus group discussions were conducted with key stakeholders, including educators, community leaders and health professionals, to explore their perceptions of the intervention’s design and implementation. These qualitative methods allowed for a deep exploration of the contextual factors that influenced the project´s acceptance and effectiveness.

The stakeholders emphasised the importance of cultural alignment in the design of the dental superheroine, highlighting how her visual and symbolic attributes resonated with local traditions and community values. For example, the incorporation of Chachapoyan iconography and use of professional dental symbols were noted as the elements that enhanced the authenticity and relatability of the superheroine. Additionally, the participants praised the interactive educational materials, describing them as engaging tools that effectively conveyed oral health messages while fostering active participation.

The focus groups also provided insights into the intervention’s impact on community dynamics. Participants reported increased awareness and more frequent discussions about oral health within their families and social circles. They attributed this shift to the participatory nature of the activities and the emotional connection created by the superheroine. These findings underscore the importance of designing culturally and contextually relevant educational interventions that prioritise community engagement and collaborative learning.

The qualitative insights gained from these discussions were instrumental in refining the implementation strategies of the project, ensuring that the dental superheroine served not only as an educational tool but also as a catalyst for encouraging collective action and shared responsibility in the promotion of oral health.

### Community dissemination activities

The public presentation of the dental superheroine was carefully planned to maximise community engagement and foster a sense of collective participation. The UNTRM institutional anniversary parade served as a platform to introduce the superheroine and disseminate key oral health messages through culturally adapted activities.

The event began with a vibrant procession, with the dental superheroine leading the parade, accompanied by groups of students from the Professional School of Stomatology dressed in traditional Chachapoyan attire and dentist costumes. This fusion of local culture with modern superhero imagery created a visual narrative that resonated with the audience. The educational messages were displayed on banners throughout the parade, highlighting the importance of oral hygiene through activities tailored to promote oral health.

Along the parade route, student groups from the School of Dentistry led hands-on brushing demonstrations using dental models, superheroine flipcharts, and interactive 3D-printed character models. These activities were designed to engage all age groups, creating an inclusive environment where the public could learn and participate actively. The event culminated in a march before the city authorities of Chachapoyas, during which a speech was delivered recognising the superheroine as a community ambassador for oral health. In this symbolic moment, the superheroine was presented as a unifying figure representing the collective effort to address oral health challenges in the region. To conclude the event, educational kits containing leaflets, toothbrushes and miniature models of the superheroine were distributed to reinforce the day’s messages and extend their impact beyond the parade. This multifaceted approach to community outreach proved effective in capturing the attention and imagination of participants, laying the groundwork for promoting sustained engagement in oral health practices. By integrating educational content with cultural celebration, the event highlighted the potential of creative, community-centred strategies to promote public health in meaningful and lasting ways.

### Study population

The 300 participants in this study were selected to provide a sample sufficiently representative of the Chachapoyas community for an initial exploratory analysis of the dental superheroine's impact on oral health promotion. Convenience sampling was used during the character’s public presentation, allowing for a diverse mix of ages, educational backgrounds and marital statuses. This approach aimed to capture a broad range of perceptions and experiences, enabling a more comprehensive understanding of how different segments of the community responded to the intervention.

This study was conducted in accordance with the ethical standards of the Universidad Nacional Toribio Rodríguez de Mendoza de Amazonas. No formal ethical approval was needed, as the university's ethics committee does not mandate approval for studies that do not involve animal experimentation, human clinical procedures, or interventions affecting health outcomes. However, in compliance with data protection regulations and ethical research practices, all collected data were anonymised to ensure participant confidentiality. No personally identifiable information was recorded or stored, and responses were processed following the data anonymisation guidelines established in the Guide to Basic Anonymization by the Personal Data Protection Commission (PDPC) of Singapore. Informed consent was obtained from all the subjects who participated in the study.

The present research used the industrial design registered under the title N°6825 The “Doll” valid until 03-01-2023 and registered before the Instituto Nacional de Defensa de la Competencia y de la Protección de la Propiedad Intelectual of Perú (INDECOPI).

### Statistical analysis of correlations

To analyse the relationship between the participants' positive perceptions of the character and their level of cultural engagement, Pearson's correlation analysis was conducted. This statistical analysis yielded a *P* < 0.05, indicating that the correlation observed between these variables is statistically significant and not due to chance. In addition, a simple linear regression model was used to assess how the levels of cultural engagement predict positive character perception. The model yielded an R^2^ = 56.1%, suggesting that the levels of cultural engagement explain a strengthening of the hypothesis that a stronger cultural connection enhances the efficacy of the character in promoting oral health behaviours.

### Measuring the initial impact of character on oral health

The impact of the persona was assessed through a structured survey designed to capture the participants' attitudes, knowledge and intentions regarding oral health after their exposure to the persona. The survey questions included Likert-scale items, where the participants rated the influence of the character on their interest and commitment to oral hygiene practices and their intention to adopt specific behaviours, such as regular brushing and mouthwash use.

The survey assessed five main dimensions: positive perception of the character, overall quality, cultural identification, social recognition and perceived impact on social networks. These indicators enabled an initial assessment of the character’s influence on oral health attitudes within the community. This approach helped to capture the immediate attitudinal impact among the study’s participants.

## Results

### Demographic profile of the sample

Table 1 presents the demographics of the 300 respondents who participated in the event. The most represented age group was 25-31 years (29.33%), followed by 32-38 years (26%). The data indicate a slight predominance of women over men, with 53% of the sample being women and 47% being men. In terms of the educational background, 48.33% reported having a university education, whereas 19.34% had a background in studies in pedagogical institutes. Only 10% had a high school education, indicating that the sample was composed mainly of people with a high level of education. With respect to marital status, 40.33% of the respondents were single, 32.67% were cohabiting, 18.33% were married and 9% reported being separated. This diversity highlights the importance of tailoring educational interventions to heterogeneous audiences.

**Table 1:** Demographics of the sample.

**Variable**	**Categories**	**Frequency**	**Percentage**
Age	18-24	52	17.34%
	25-31	88	29.33%
	32-38	78	26.00%
	39-45	50	16.67%
	46-52	31	10.33%
	53-59	1	0.33%
Sex	Female	159	53.00%
	Male	141	47.00%
Education level	Pedagogical	58	19.34%
	Secondary	30	10.00%
	Technical	67	22.33%
	University	145	48.33%
Marital status	Single	121	40.33%
	Cohabiting	98	32.67%
	Married	55	18.33%
	Separated	26	8.67%
Total		300	100%

### Perception of and commitment to the dental superheroine

Table 2 presents the results of the survey assessing the participants’ perceptions of the dental superheroine and their level of engagement with the campaign. The majority of respondents (82%) reported a high level of positive perception, indicating a strong acceptance of the character within the community. Furthermore, the initiative received an overwhelmingly positive evaluation from 89.67%, thereby underscoring the favourable community response and validating the cultural resonance of the character.

**Table 2:** Perception levels.

**Variable/Dimensions**	**Level**	**Frequency**	**Percentage**
Positive perception	High	246	82.00%
	Medium	53	17.67%
	Low	1	0.33%
Overall rating	High	269	89.67%
	Medium	29	9.66%
	Low	2	0.67%
Cultural identification	Strong	77	25.67%
	Moderate	213	71.00%
	Mild	10	3.33%
Social recognition	Strong	231	77.00%
	Moderate	67	22.33%
	Mild	2	0.67%
Perceived social media impact	High	45	15.00%
	Medium	211	70.33%
	Low	44	14.67%
Total		300	100%

However, the results revealed moderate levels of cultural identification and perceived impact on social networks. A mere 25.67% of the respondents reported a strong cultural connection to the character, whereas 70.33% expressed a moderate connection. The findings suggest that while a character resonates with the local culture, there is potential to deepen its alignment with specific cultural symbols and narratives. In terms of the social media impact, only 15% of the respondents perceived a high level of influence, whereas 70.33% rated it as moderate. These findings underscore the need to refine digital dissemination strategies to amplify the character’s presence on social platforms and to engage younger, tech-savvy demographics.

### Cultural engagement and correlation with positive perception

Table 3 presents an analysis of three variables related to culture and local participation: popular culture, cultural influence and participation in local, media and charity events. Regarding popular culture, the majority of the population reported a high level of engagement (55.66%), followed by a medium level (42.67%) and a marginally low level (1.67%). This suggests that most participants are familiar with key cultural elements. In contrast, cultural influence showed a dominant presence of medium levels (64.33%), indicating that most people experience or exert a moderate level of cultural influence. High levels were reported by 34% of respondents, with low levels remaining minimal (1.67%).

**Table 3:** Popular culture.

**Variable**	**Level**	**Frequency**	**Percentage**
Popular culture	High	167	55.66%
	Medium	128	42.67%
	Low	5	1.67%
Cultural influence	High	102	34.00%
	Medium	193	64.33%
	Low	5	1.67%
Participation in local, media and charity events	High	202	67.33%
	Medium	91	30.34%
	Low	7	2.33%
Total		300	100%

Participation in local, media and charity events was notably high, with 67.33% of respondents reporting strong involvement. This reflects a community-oriented culture in Chachapoyas, which likely enhances the receptiveness of the population to educational initiatives like the dental superheroine. These findings collectively underscore the importance of embedding educational interventions within the local cultural context to maximise engagement.

Table 4 further illustrates that high levels of popular culture are predominantly associated with positive perceptions (51.66%), whereas lower levels have a marginal impact. This finding reinforces the critical role of cultural alignment in shaping favourable educational outcomes. Medium levels of popular culture show an incremental transition toward higher positive perceptions (30.33%), suggesting the potential for further improvement through targeted cultural integration strategies.

**Table 4:** Popular culture level and positive perception level.

**Level of positive perception**							**Row total**
Popular culture level	High perception	%	Moderate perception	%	Mild perception	%	
High	155	51.66	12	4.00	0	0.00	167
Medium	91	30.34	37	12.34	0	0.00	128
Low	0	0.00	4	1.33	1	0.33	5
Column total	246		53		1		300

### Statistical analysis and significance of the correlation

A Pearson correlation analysis was performed to evaluate the relationship between the participants’ levels of cultural engagement and their positive perceptions of the character. A *P* < .05 was obtained, indicating a statistically significant correlation. This suggests that a higher level of cultural engagement is associated with a more favourable perception of the character.

**Fig. 2:**
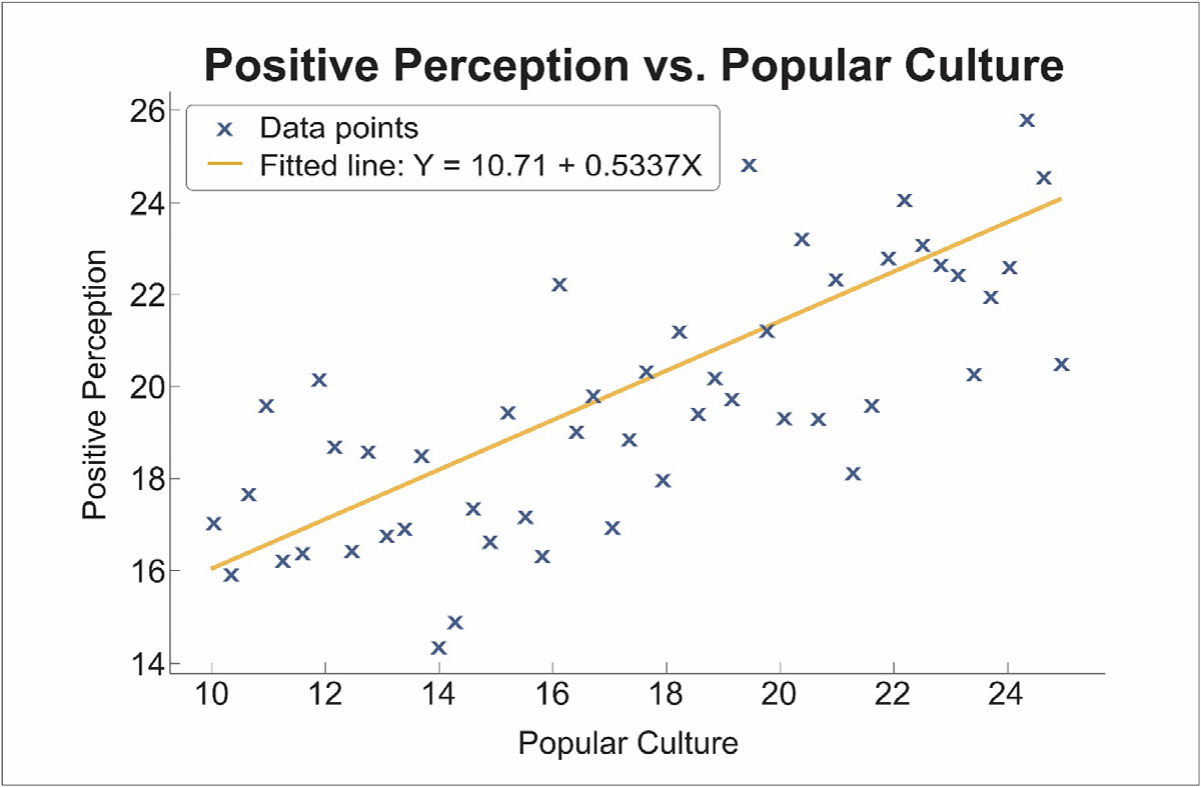
Correlation between cultural engagement and positive perception of the dental superheroine.

The R^2^ = 56.1% indicates that more than half of the variability in the participants’ positive perceptions of the dental superheroine can be attributed to their level of cultural engagement. This relationship is illustrated in Figure 2, where a positive linear trend is evident, confirming the strength of the association. This finding underscores the importance of incorporating culturally relevant elements into educational resources. Through the alignment of the attributes of the superheroine with the cultural values and symbols familiar to the community, the initiatives successfully fostered deeper connections and engagements. These findings emphasise the need to integrate cultural frameworks into educational strategies to ensure resonance and impact, particularly in a context where cultural identity plays a pivotal role in community dynamics.

These findings align with prior research demonstrating the effectiveness of culturally tailored educational tools. For example, studies by Heaton et al.^23^ underscore the pivotal role of culturally grounded storytelling in promoting health knowledge among indigenous communities, whereas Kreindler et al.^11^ highlight the importance of aligning health campaigns with the local cultural symbols to enhance public reception. Similarly, the findings resonate with Dubourg and Baumard,^9^ who argue that the cultural familiarity in the fictional characters amplifies message retention and emotional engagement.

The success of the dental superheroine aligns with these observations, underscoring the universal applicability of culturally resonant educational strategies. The high level of positive perception linked to cultural engagement further underpins the model’s adaptability to other rural or culturally distinct settings. However, the moderate ratings for digital impact suggest that while cultural alignment is critical, integrating digital strategies tailored to younger audiences could complement and extend the reach of the intervention.

By integrating cultural values with educational goals, this study not only reinforces existing literature but also sets a precedent for leveraging cultural engagement as a cornerstone for impactful educational interventions in underserved communities.

### Qualitative analysis: A pedagogical and cultural perspective

The qualitative component of this study offered valuable insights into how the dental superheroine was perceived and how well it aligned with community values. Interviews with key community leaders, educators and participants revealed recurring themes: strong cultural resonance, engagement through creative elements and the need for improved digital dissemination.

Respondents consistently emphasised the superheroine’s symbolic connection to Chachapoyan traditions, particularly through visual elements inspired by local icons such as Matiaza Rimachi. As one community leader stated, ‘This character feels like one of us; she speaks to our values and traditions while promoting something as important as oral health.’ Such feedback highlights the importance of incorporating culturally familiar motifs to increase acceptance and relevance.

Participants expressed deep emotional and cultural connections with the character. A local teacher remarked, ‘The character feels like it was created for us, reflecting not just health messages but also the essence of our traditions.’ Another community member added, ‘Her shield and colours remind me of our region’s history and resilience.’ These comments highlight the effectiveness of weaving local symbols and narratives into health education campaigns.

Younger participants found the interactive elements particularly engaging. One student noted, ‘Colouring the 3D model was fun, and it made me think more about brushing my teeth.’ These insights demonstrate the value of participatory and hands-on learning activities in improving message retention and fostering behavioural change.

### Emerging themes

The superheroine’s alignment with local traditions was frequently cited as a strength. The use of traditional symbols and colours was met with positive feedback, fostering a sense of familiarity and pride among the participants. However, some respondents suggested that deeper integration of regional attention or historical references could enhance the authenticity.

The integration of visual materials, interactive activities and storytelling proved to be a potent combination. The respondents expressed appreciation for the methods’ capacity to render oral health education approachable and memorable. A recurrent recommendation was the incorporation of more personalised storytelling, incorporating local myths or legends.

While the campaign achieved success in the physical setting, its digital presence was perceived as deficient. A community youth leader articulated this sentiment, stating, ‘The superheroine could have reached more people if there were more posts and videos on social media.’ This feedback underscores the need for strategic digital content to complement face-to-face interventions.

The qualitative insights align with the quantitative findings, particularly the strong correlation between cultural engagement and positive perceptions. The high R^2^ value (56.1%) underscores the significance of cultural alignment, as evidenced by the participants’ narratives regarding the character’s relatability and impact. Furthermore, the proposal to enhance digital strategies aligns with the moderate scores for perceived social media impact (15% high impact).

By integrating these qualitative themes with quantitative data, this study demonstrates how culturally resonant educational tools can bridge emotional and cognitive gaps, fostering greater acceptance and promoting behavioural change.

## Discussion

This study underscores the potential of culturally relevant fictional characters as educational tools to promote oral health habits in rural communities. The introduction of a dental superheroine in Chachapoyas has demonstrated that health messages, when embedded in a culturally meaningful framework, can effectively engage community interest and foster commitment to health practices. Integrating the character’s educational function with its cultural relevance presents an innovative strategy for promoting health in underserved populations, effectively transcending the conventional barriers of health education. The educational strategies employed in this study are firmly grounded in constructivist pedagogy, which emphasises the active role of learners in building their own knowledge through meaningful interaction with their environment. The use of a dental superheroine as the central figure aligns with constructivist principles by creating a culturally resonant and participatory learning experience. Through storytelling, interactive materials and culturally familiar symbols, the intervention fostered an engaging learning environment that encouraged participants to internalise key health messages.

A core tenet of constructivism is that knowledge is most effectively acquired when learners actively engage with content in personally meaningful ways. The superheroine served as an interactive platform for this engagement, particularly through hands-on activities such as colouring 3D models and interacting with visual materials. These activities not only captured participants’ attention but also encouraged them to link their cultural identity with health-promoting behaviours. For instance, the use of a shield symbolising protection, drawn from local traditions, created a powerful connection between familiar cultural symbols and new oral health knowledge.

The distribution of white PLA prototypes for attendees to colour exemplifies the practical application of constructivist principles. This hands-on activity transformed abstract concepts of oral hygiene into tangible, engaging experiences that were both educational and enjoyable. Similarly, community discussions held during the superheroine presentation encouraged meaningful dialogue, allowing participants to connect health messages to their personal experiences and cultural backgrounds. These examples demonstrate how active participation can improve knowledge retention and foster a sense of ownership over the learning process.

This model holds significant promise for broader application in educational settings, particularly those serving culturally diverse or underserved populations. The adaptability of the dental superheroine to reflect local cultural values suggests that similar interventions could be customised for other communities by incorporating region-specific narratives and symbols. Furthermore, this approach promotes the development of critical competencies such as cultural awareness and autonomous learning by empowering participants to view themselves as active agents in their own health and education.

Previous studies have established the educational value of fictional characters, asserting their ability to enrich the learning experience by fostering interaction and creativity in children.^24^^,^^25^ In line with these findings, the dental superheroine offers a versatile educational symbol that can be incorporated into classrooms through text production, videos and school campaigns to promote oral health awareness. For preschool children, prototypes made of white PLA can be distributed for colouring activities, in line with the constructivist approach prevalent in rural Andean schools.^26^ This participatory method not only supports learning but also fosters a sense of ownership and engagement among young learners.

To contextualise the study, qualitative data were collected through interviews and open-ended survey responses that provided a humanised perspective on participants' experiences. These accounts highlight the cultural resonance and educational impact of the superheroine while also identifying areas for improvement.

The participants often emphasised the cultural significance of the superheroine. A dentistry student commented, ‘The character feels like it was created for us, reflecting not only health messages but also the essence of our traditions.’ Similarly, an oral rehabilitation teacher noted, ‘Her shield reminds me of the protective symbols we have in our local folklore. It connects well with our values.’ These comments demonstrate how the character’s design and symbolism resonated deeply with the community.

Younger participants also found the character engaging and relatable. A middle school student shared, ‘Colouring the model was fun, and it made me think more about brushing my teeth regularly.’ Such interactive elements not only entertain but also reinforce positive health behaviours, exemplifying constructivist learning in action.

While the character was met with widespread approval, certain participants identified areas that necessitated enhancement. For example, a community leader suggested, ‘Including more traditional attire could make the character even more relatable to older generations.’ Another common critique was the moderate impact on social media. A youth participant commented, ‘I think more Instagram stories or videos could have made the campaign more exciting for people my age.’ These insights highlight the need for a balanced approach that caters to both traditional and digital audiences.

The dental superheroine exemplifies an innovative intersection of public health and pedagogy, demonstrating how interdisciplinary approaches can address complex societal issues. By integrating health promotion with educational strategies, the initiative achieved a dual impact: raising awareness about oral hygiene while cultivating critical learning skills among participants. This aligns with global trends that emphasise holistic approaches to education and public health interventions.

Similar international initiatives, such as the use of animated characters to promote hygiene in Tanzania^13^ or nutrition education in Brazil,^12^ have demonstrated that culturally tailored fictional figures can effectively bridge gaps in knowledge and engagement. The dental superheroine builds on this legacy by offering a scalable approach.

The findings of this study have broader implications, particularly in regions with limited access to health education. Embedding health messages within culturally resonant narratives can be replicated and adapted to diverse communities worldwide. For instance, incorporating local folklore or historical figures into health campaigns has been shown to enhance both relatability and effectiveness, as evidenced by successful interventions in a range of rural and urban settings globally. These results highlight the universal potential of combining cultural identity with educational innovation. The strong correlation between cultural engagement and positive perceptions of the character underscores the importance of embedding cultural values into health promotion strategies. This is consistent with existing research in health communication, which shows that characters rooted in cultural identity enhance message retention and acceptance.^27^^,^^28^ Fictional figures are especially effective in shaping health behaviours when they reflect the social and cultural values of their target communities.^29^^,^^30^ In this context, the dental superheroine functions as a culturally resonant figure, capable of generating both immediate engagement and sustained interest in oral health. As Breuer, Lindow and Betsch^31^ argue, reusing culturally aligned characters across multiple campaigns can amplify their long-term impact. The positive reception of the character, demonstrated by high perception scores, highlights its potential to strengthen community identity and support health initiatives. Similar approaches using superheroes or historical figures in health campaigns have proven effective in leveraging cultural symbolism to improve health behaviours.^32^^,^^33^^,^^34^ The findings of this study align with these insights and suggest that fictional characters, such as the dental superheroine, can successfully integrate health messages into the cultural fabric of communities with strong local identities.

Despite the absence of traditional regional attire in character design, the use of professional colours and the emblem of the Escuela Professional de Estomatología de la Universidad Nacional Toribio Rodríguez de Mendoza resonated with community values. This professional symbolism appears to be a crucial factor in fostering community acceptance. This emotional connection, as highlighted in studies on Olympic mascots, fosters engagement and alignment with community identity.^35^ Additionally, the character’s ability to counter external cultural influences and stereotypes further demonstrates its value as a culturally adaptive tool.^36^^,^^37^

Although the campaign's digital outreach achieved moderate success, the relatively low perceived impact on social media points to opportunities for improvement. Enhancing the character’s digital presence through live streaming, interactive content, or gamified storytelling could significantly boost engagement, particularly among younger and more digitally connected audiences. Research shows that storytelling is a powerful instrument in health promotion, capable of fostering emotional bonds and sustaining attention over time.^38^, ^39^, ^40^

Further integration of traditional symbols and narratives could deepen cultural identification and embed health messages more effectively within community identity. The superheroine subtly echoes prominent local female figures, such as Matiaza Rimachi and Virgen Asunta, thereby fostering emotional and cultural connections. This strategy parallels global practices that embed fictional characters within local narratives to increase cultural relevance.^41^^,^^42^

This study presents a replicable model for culturally responsive health promotion that could benefit rural and culturally diverse communities worldwide. By breaking the fourth wall and focusing on communities directly, as suggested by Bausela,^43^ such initiatives can promote lasting behavioural changes. Policymakers and health professionals should consider the integration of fictional characters into health campaigns as a way to encourage community participation and foster meaningful, long-term public health outcomes.^44^ This culturally adapted approach aligns with recent global recommendations, such as the Bangkok Declaration on Oral Health, which urges the integration of oral health into national public health agendas and calls for equitable, culturally responsive care strategies at all levels of society.^45^

### Clinical relevance of the dental superheroine

The implementation of the dental superheroine as a culturally adapted educational tool extends beyond community engagement; it holds potential clinical significance. By enhancing oral health literacy through relatable storytelling and symbolic association, the superheroine promotes early preventive behaviours, better oral hygiene practices and greater adherence to dental recommendations. This shift in behaviour can contribute to a measurable reduction in the prevalence of common oral diseases such as dental caries and periodontal disease, particularly in underserved rural populations. Additionally, the superheroine helps to foster a more positive patient–provider relationship by reducing anxiety in paediatric patients and strengthening communication between dental professionals and community members. From a public health perspective, the character serves as a behavioural intervention aligned with preventive care goals, supporting continuity of care and empowering individuals to take an active role in maintaining their oral health.

This study has some limitations that should be considered when the findings are interpreted. First, even though the sample is representative of event participants, it may not fully capture the broader demographic profile of Chachapoyas, which could affect the generalisability of the findings. In addition, the cross-sectional design limits conclusions about the long-term impact of character on actual health behaviours. Future studies could address these limitations by expanding the sample size and using longitudinal designs to assess sustained changes in oral health habits over time. In addition, the effectiveness of digital storytelling has not been examined, nor have comparative studies been conducted with other culturally adapted educational models in their context.

This study demonstrated that fictional characters based on local cultural elements offer a promising and adaptable approach to health promotion, especially in regions with strong cultural identities. The dental superheroine serves as an innovative model for combining entertainment and education, setting a precedent for culturally integrated public health strategies. It also contributes to a theoretical understanding of how fictional characters can enhance the effectiveness of health messages and provides a creative framework for addressing health challenges in culturally diverse communities.

### Educational implications

By integrating qualitative and quantitative findings, this study underscores the value of combining cultural resonance with interactive and participatory educational strategies. The participants’ insights not only validate the approach but also provide actionable recommendations for future campaigns, such as incorporating more traditional elements and expanding the digital outreach. These adaptations could further enhance the model’s effectiveness and applicability to other culturally diverse settings.

### Broader educational and social implications

Beyond its clinical relevance, the dental superheroine represents a transdisciplinary strategy that merges and integrates health education, cultural identity and community empowerment. As a pedagogical tool, the character uses narrative-based learning to promote long-term behavioural changes through symbolic association and cultural resonance. Its visual and conceptual design aligns with constructivist educational models, fostering greater engagement among participants who may otherwise be excluded from conventional health promotion. Additionally, the superheroine contributes to the reinforcement of local identity and collective pride, particularly in underserved populations where cultural representation in health campaigns is often limited. This approach not only enhances oral health literacy but also paves the way for innovation in health communication, including the development of gamified learning tools and culturally grounded digital interventions. By bridging art, psychology and public health, the intervention offers a replicable model for future community-based strategies in diverse cultural contexts.

## Conclusion

This study demonstrates the potential of culturally resonant fictional characters as effective tools for health promotion, as exemplified by the introduction of a dental superheroine in Chachapoyas, Perú. By embedding oral health messages within a character that reflects community values, this strategy effectively increased awareness while strengthening the connection to local cultural identity. These findings underscore the transformative potential of culturally tailored interventions, particularly in underserved or rural areas where conventional health education efforts often fail to resonate.

The dental superheroine model offers a scalable and adaptable framework for integrating public health initiatives into culturally relevant contexts. This character-driven approach presents an innovative alternative to traditional methods by leveraging familiar cultural symbols and interactive storytelling to promote meaningful behaviour change. Policymakers and health professionals can adopt similar strategies in diverse settings to enhance community engagement and support sustainable health outcomes.

While this study focused primarily on the short-term effects of the superheroine on public perceptions and attitudes, it lays the groundwork for future research on long-term behavioural changes. Evaluating the durability of these effects through longitudinal studies could provide deeper insights into the sustainability of such culturally resonant strategies. In addition, incorporating digital tools, such as gamification and interactive media, could further enhance the reach, engagement and effectiveness of similar health promotion campaigns.

The dental superheroine serves as a compelling model for delivering both education and entertainment to address public health challenges in culturally diverse communities. Its success underscores the value of combining cultural resonance with participatory and constructivist educational principles, offering a creative and adaptable framework for advancing health equity and improving the quality of life at both local and global levels.

## Conflict of interests

None declared.
